# Bioinformatic analysis of key pathways and genes involved in pediatric atopic dermatitis

**DOI:** 10.1042/BSR20193517

**Published:** 2021-01-06

**Authors:** Tianyi Wang, Bingxin Zhang, Danhui Li, Xiaoli Qi, Chijin Zhang

**Affiliations:** 1 Department of Dermatology, First Teaching Hospital of Tianjin University of TCM, China; 2 School of Pharmacy, University of Auckland, Auckland, New Zealand; 3 Department of Pediatric Dermatology, Tianjin Academy of Traditional Chinese Medicine Affiliated Hospital, China

**Keywords:** Bioinformatics analysis, Differentially expressed gene, Microarray, Pediatric atopic dermatitis

## Abstract

The initiation of atopic dermatitis (AD) typically happens very early in life, but most of our understanding of AD is derived from studies on AD patients in adult. The aim of the present study was to identify gene signature speficic to pediatric AD comapred with adult AD. The gene expression profiles of four datasets (GSE32924, GSE36842, GSE58558, and GSE107361) were downloaded from the GEO database. Gene ontology (GO) and Kyoto Encyclopedia of Genes and Genomes pathway (KEGG) enrichment analyses were performed, and protein–protein interaction (PPI) network was constructed by Cytoscape software. Total 654 differentially expressed genes (DEGs) (394 up-regulated and 260 down-regulated) were identified in pediatric AD samples with adult AD samples as control. The up-regulated DEGs were significantly enriched in the migration and chemotaxis of granulocyte and neutrophil, while down-regulated DEGs were significantly enriched in biological adhesion. KEGG pathway analysis showed that up-regulated DEGs participated in chemokine signaling pathway while down-regulated DEGs participated in adherens junction, focal adhesion, and regulation of actin cytoskeleton. The top 10 hub genes GAPDH, EGFR, ACTB, ESR1, CDK1, CXCL8, CD44, KRAS, PTGS2, and SMC3 were involved in chemokine signaling pathway, cytokine–cytokine receptor interaction, interleukin-17 signaling pathway, and regulation of actin cytoskeleton. In conclusion, we identified DEGs and hub genes involved in pediatric AD, which might be used as therapeutic targets and diagnostic biomarkers for pediatric AD.

## Introduction

Atopic dermatitis (AD) is the most common inflammatory skin disease with an estimated prevalence of around 20% in children and 7–10% in adults [1–4]. AD is predominantly a Th2/Th22 polarized disease with Th1 polarization in the chronic phase and the impairment of Th17 pathway [5]. The initiation of AD typically happens very early in life, but most of our understanding of AD is derived from studies on AD patients in adult. Therefore, the molecular mechanism underlying pediatric AD initiation and progression is elusive, resulting in a lack of specific treatment for this disease.

Bioinformatics analysis of microarray data is increasingly valued as a promising tool in gene expression profiling in inflammatory diseases to identify differentially expressed genes (DEGs) that play important role in the diseases [6–8]. However, comparative analysis of the DEGs between pediatric AD and adult AD remains to be elucidated.

The aim of the present study was to explore gene signature of pediatric AD and identify differentially expressed genes involved in pediatric AD comapred with adult AD. In present study, we download the original data (GSE32924, GSE36842, GSE58558, and GSE107361) from Gene Expression Omnibus and compared gene expression profiles of pediatric AD with those in adult AD. The DEGs were identified and analyzed by gene ontology (GO) and pathway enrichment analysis.

## Materials and methods

### Identification of DEGs

From the Gene Expression Omnibus (http://www.ncbi.nlm.nih.gov/geo/), four gene expression profiles (GSE32924, GSE36842, GSE58558, and GSE107361) were selected because they were on gene expression profiling of AD samples (total 49 adult AD samples versus 19 pediatric AD samples) based on Affymetrix GPL570 platform [9–12]. The original probe-level data were converted into gene-level data using Robust multi-array average (RMA) approach for background correction and normalization. Next, limma packagein R language was used to identify the DEGs between pediatric and adult samples. Subsequently, a between-subjects *t*-test was performed to identify DEGs of each AD group with the cutoff criteria of log2 fold change (FC) >2 and FDR <0.01. Volcano plots were generated to visualize the distribution of DEGs between pediatric and adult samples of AD patients.

### Gene ontology and pathway enrichment analysis of DEGs

Bioinformatics analysis of the DEGs was performed as described previously [13].

Gene ontology (GO) and Kyoto Encyclopedia of Genes and Genomes (KEGG) analysis were performed by employing an online software DAVID Database (https://david.ncifcrf.gov/). *P*<0.05 was considered statistically significant.

### Integration of protein–protein interaction network

STRING online database (http://string-db.org) was used for analyzing the protein–protein interaction (PPI) information. The cut-off criteria were a combined score of > 0.4 for a PPI network and a node degree of > 10 for screening hub genes. Cytoscape MCODE plug-in was used for searching clustered sub-networks. The default parameters were as follows: degree cutoff ≥10, node score cutoff ≥0.2, *K*-core ≥2, and max depth = 100.

## Results

### Identification of DEGs

**Figure 1:**
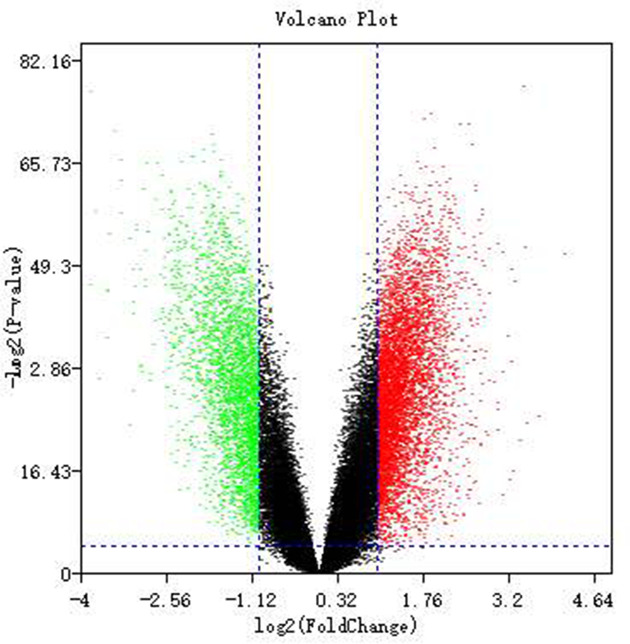
Volcano plots of genes that are significantly different between pediatric and adult controls The *X*-axis indicates the *P* values (log scale), whereas the *Y*-axis shows the fold change (log scale). Each symbol represents a different gene, and the red/green color of the symbols categorize the up-regulated/down-regulated genes falling under different criteria (*P*-value and fold change threshold). *P*-value <0.01 is considered as statistically significant, whereas fold change = 2 is set as the threshold.

**Figure 2:**
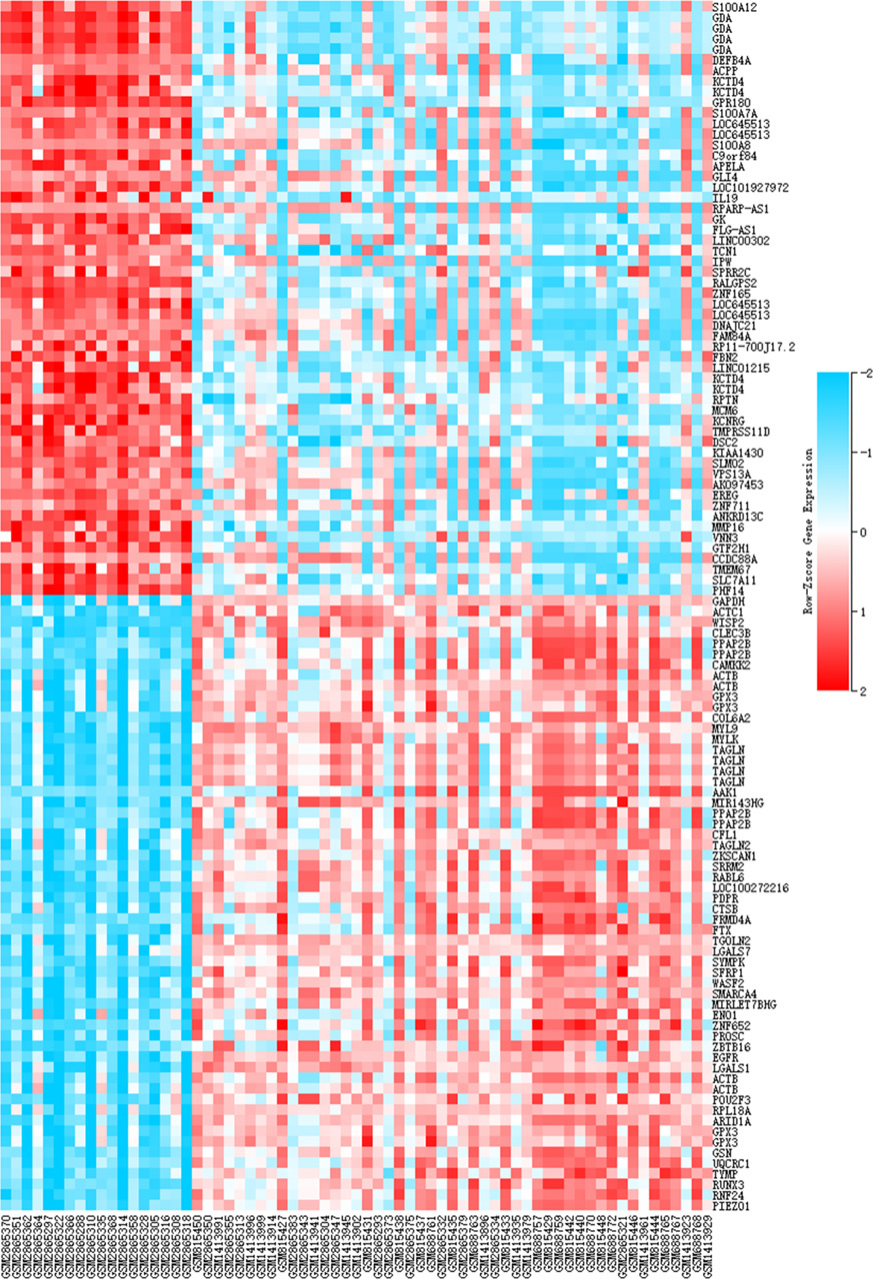
Heat map of the top 100 differentially expressed genes Shown were 50 up-regulated genes and 50 down-regulated genes). Each column represented a biological sample and each row in the heat map represents a gene. Red: up-regulation; Blue: down-regulation.

A total of 654 genes (394 were up-regulated and 260 were down-regulated) special to pediatric AD samples were identified after the analyses in all four independent cohorts with adult AD sampels as control (Supplementary Tables S1 and S2). Red or green dots in the volcano plots represented significantly up- or down-regulated genes, respectively (Figure 1). The top 50 up- and down-regulated genes were shown in the heat map (Figure 2).

### Functional and pathway enrichment analyses

We uploaded all DEGs to the online software DAVID to identify over-represented GO categories and KEGG pathways. GO term enrichment analysis showed that up-regulated DEGs were significantly enriched in the migration and chemotaxis of granulocyte and Neutrophil, while down-regulated DEGs were mainly involved in a multi-organism process, In addition, molecular function analysis showed that up-regulated DEGs were mainly associated with chemokine activity, while down-regulated DEGs were involved in protein binding (Table 1). Furthermore, KEGG pathway analysis showed that up-regulated DEGs participated in the chemokine signaling pathway while down-regulated DEGs participated in adherens junction, focal adhesion, and regulation of actin cytoskeleton (Table 2).

**Table 1:** Gene ontology analysis of DEGs associated with pediatric AD

Category	Term	Involved in	n^*^	%	*P*
Up-regulated					
GOTERM_BP_FAT	GO:0097530	granulocyte migration	9	2.3	1.32E-03
GOTERM_BP_FAT	GO:0006275	regulation of DNA replication	9	2.3	1.56E-03
GOTERM_BP_FAT	GO:1990266	neutrophil migration	8	2.0	1.86E-03
GOTERM_BP_FAT	GO:0071621	granulocyte chemotaxis	8	2.0	3.13E-03
GOTERM_BP_FAT	GO:0030593	neutrophil chemotaxis	7	1.8	4.78E-03
GOTERM_CC_FAT	GO:0005615	extracellular space	40	10.2	9.77E-03
GOTERM_CC_FAT	GO:0098687	chromosomal region	13	3.3	2.74E-02
GOTERM_MF_FAT	GO:0005125	cytokine activity	13	3.3	1.03E-03
GOTERM_MF_FAT	GO:0042379	chemokine receptor binding	7	1.8	1.16E-03
GOTERM_MF_FAT	GO:0008009	chemokine activity	6	1.5	2.20E-03
GOTERM_MF_FAT	GO:0016791	phosphatase activity	12	3.1	1.71E-02
GOTERM_MF_FAT	GO:0016810	hydrolase activity, acting on carbon–nitrogen bonds	8	2.0	2.12E-02
Down-regulated					
GOTERM_BP_FAT	GO:0016032	viral process	32	12.3	4.97E-06
GOTERM_BP_FAT	GO:0044764	multiorganism cellular process	32	12.3	5.75E-06
GOTERM_BP_FAT	GO:0022610	biological adhesion	46	17.7	5.76E-06
GOTERM_BP_FAT	GO:0044403	symbiosis, encompassing mutualism through parasitism	32	12.3	9.55E-06
GOTERM_BP_FAT	GO:0044419	interspecies interaction between organisms	32	12.3	9.55E-06
GOTERM_CC_FAT	GO:0005912	adherens junction	37	14.2	8.19E-12
GOTERM_CC_FAT	GO:0070161	anchoring junction	37	14.2	1.64E-11
GOTERM_CC_FAT	GO:0070062	extracellular exosome	73	28.1	5.49E-08
GOTERM_CC_FAT	GO:1903561	extracellular vesicle	73	28.1	6.76E-08
GOTERM_CC_FAT	GO:0043230	extracellular organelle	73	28.1	6.86E-08
GOTERM_MF_FAT	GO:0008092	cytoskeletal protein binding	31	11.9	1.30E-06
GOTERM_MF_FAT	GO:0032403	protein complex binding	29	11.2	1.70E-06
GOTERM_MF_FAT	GO:0050839	cell adhesion molecule binding	19	7.3	5.91E-05
GOTERM_MF_FAT	GO:0044877	macromolecular complex binding	36	13.8	7.47E-05
GOTERM_MF_FAT	GO:0098641	cadherin binding involved in cell-cell adhesion	14	5.4	1.70E-04

* Number of enriched genes in each term. If there were more than five terms enriched in this category, the top five terms based on *P* value were chosen.

**Table 2:** KEGG pathway analysis of DEGs associated with AD

Category		Term	Count^*^	%	*P*
Up-regulated					
KEGG_PATHWAY	hsa04062	Chemokine signaling pathway	8	2.0	0.044
KEGG_PATHWAY	hsa04060	Cytokine–cytokine receptor interaction	9	2.3	0.061
KEGG_PATHWAY	hsa04064	NF-kappa B signaling pathway	5	1.3	0.065
KEGG_PATHWAY	hsa04012	ErbB signaling pathway	5	1.3	0.065
KEGG_PATHWAY	hsa05323	Rheumatoid arthritis	5	1.3	0.067
Down-Regulated					
KEGG_PATHWAY	hsa04520	Adherens junction	6	2.3	0.004
KEGG_PATHWAY	hsa04510	Focal adhesion	9	3.5	0.013
KEGG_PATHWAY	hsa04810	Regulation of actin cytoskeleton	9	3.5	0.015
KEGG_PATHWAY	hsa04530	Tight junction	5	1.9	0.044
KEGG_PATHWAY	hsa04512	ECM–receptor interaction	5	1.9	0.044

* Count: the number of enriched genes in each term. If there were more than five terms enriched in this category, the top five terms based on *P* value were chosen.

### Protein–protein interaction network construction and analysis of modules

**Figure 3:**
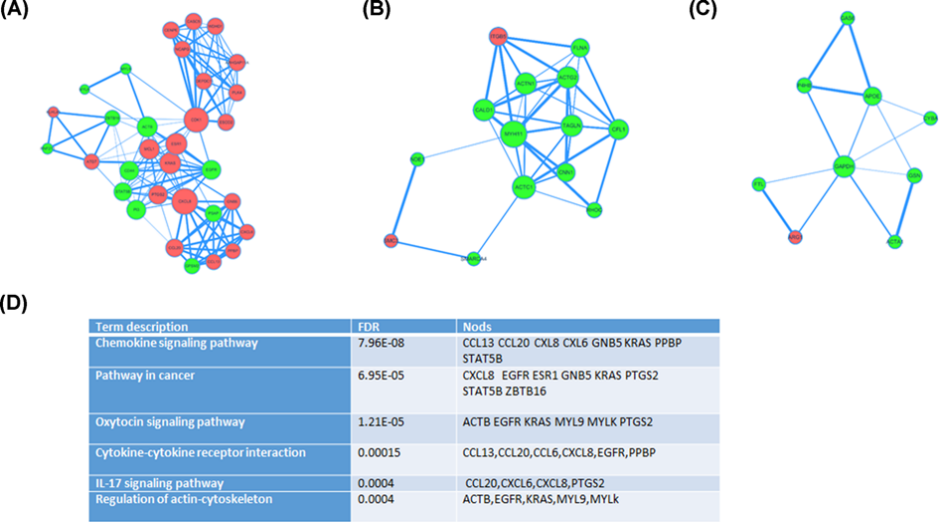
Top 3 modules from the protein-protein interaction network (**A**) module 1, (**B**) module 2, (**C**) module 3. Red: up-regulation; green: down-regulation. (**D**) the enriched pathways of the three modules (FDR< 0.0005).

Based on the information in the STRING database, the top 10 hug nodes with higher degrees were screened (Table 3). Among these nodes, GAPDH showed the highest degree. A total of 594 nodes and 1651 edges were analyzed using plug-ins MCODE. The top 3 significant modules were selected, the functional annotation of the protein involved in the modules was summarized. Enrichment analysis showed that the proteins in modules 1–3 were mainly associated with the chemokine signaling pathway, Pathway in cancer, Oxytocin signaling pathway (Figure 3).

**Table 3:** The top 10 hug nodes in protein–protein interaction network

Hub node	Information	Degree
GAPDH	Glyceraldehyde-3-phosphate dehydrogenase	89
EGFR	Epidermal growth factor receptor	69
ACTB	Actin, cytoplasmic 1	51
ESR1	Estrogen receptor	46
CDK1	Cyclin-dependent kinase1	44
CXCL8	Interleukin-8	43
CD44	CD44 antigen	41
KRAS	GTPase Kras	36
PTGS2	Prostaglandin G/H synthase 2	33
SMC3	Structural maintenance of chromosomes protein 3	27

## Discussion

Understanding of the molecular mechanism of pediatric AD might help develop approaches that can prevent atopic diathesis [14]. Previous studies have compared gene expression profiling of pediatric AD samples with adult AD sampels or normal healthy samples, respectively, but the sample size of the individual study was limited and the conclusion was controversial [9–12]. Therefore, in the present study we retrieved gene expression data of 19 pediatric AD samples and 49 adult AD samples from previous studies and identified 654 DEGs in pediatric AD samples, among which 394 were up-regulated and 260 were down-regulated. Cumulative evidence has demonstrated that the co-expressed genes normally consist of a group of genes with similar expression profiles and participate in parallel biological process. To better understand the interactions of DEGs, we further performed GO, KEGG pathway, and PPI network analysis.

GO analysis showed that DEGs mainly participated in extracellular space, anchoring junction and adherens junction, involved in granulocyte and neutrophil migration, performed functions of cytokine activity, chemokine receptor binding, chemokine activity, and cytoskeletal protein binding. Furthermore, enriched KEGG pathways of up-regulated DEGs included chemokine signaling pathway and cytokine–cytokine receptor interaction, and those of down-regulated DEGs included adherens junction, focal adhesion, and regulation of actin cytoskeleton. Therefore, all these pathways could contribute to the pathogenesis of pediatric AD.

The analysis based on PPI networks indicated that GAPDH, EGFR, ACTB showed the highest betweenness and belonged to crucial modules of the PPI network. GAPDH is a classic glycolytic enzyme involved in membrane transport and membrane-fusion, microtubule assembly, nuclear RNA export, protein phosphotransferase/kinase reactions, and translational control of gene expression [15]. The β-actin cytoskeleton functions in cellular shape and anchorage where transmembrane glycoproteins link fibronectin in the extracellular matrix with actin microfilaments on the cytoplasmic side of the membrane [16]. While GAPDH and β-actin are regarded as housekeeping genes, accumulating evidence has suggested their mRNA levels vary with cellular proliferation [17–21]. Moreover, their transcription is up-regulated rapidly in response to mitogenic stimuli including epidermal growth factor, transforming growth factor-β, and platelet-derived growth factor [22–24]. We hypothesized that β-actin and GAPDH expression levels in AD were variable and not suitable for normalizing mRNA levels. Our results were similar to some studies in asthma, which was part of the atopic march [25].

Epidermal growth factor receptor (EGFR) is a large transmembrane glycoprotein with ligand-induced tyrosine kinase activity [26]. Inhibition of EGFR signaling leads to decreased expression of cytoskeleton proteins such as actin-binding protein ACTN1 (actinin-1), increased keratinocyte adhesion, resulting in the inhibition of the migration of keratinocytes from the basal layer to the stratum corneum [27–30]. Blockade of EGFR signaling can regulate the expression of CCL26/eotaxin-3 in primary keratinocytes in AD [31,32].

In summary, we identified genes differentially expressed in pediatric AD compared with adult AD and explored their potential function and relevant pathways in the pathogenesis of pediatric AD. Moreover, our study suggested that chemokine pathway and cytoskeletal protein binding play a vital role in the molecular mechanism of pediatric AD. However, the present study has limitation because it is based on bioinformatic analysis of online datasets and the differentially expressed genes in pediatric AD should be validated by real-time PCR analysis and function assay. In particular, further studies are needed to validate GAPDH, EGFR and ACTB, which can be considered as crucial genes involved in pediatric AD, with the potential to be used in the diagnosis and therapy.

## Supplementary Material

Supplementary Tables S1-S2

## Data Availability

All data are available upon request.

## Abbreviations

AD: atopic dermatitis
DEG: differentially expressed gene
EGFR: epidermal growth factor receptor
GO: Gene ontology
KEGG: Kyoto Encyclopedia of Genes and Genomes
PPI: protein–protein interaction
RMA: Robust multi-array average
