# Supplementary material for: Bioinformatic analysis of key pathways and genes involved in pediatric atopic dermatitis
Source: Biosci Rep. 2021 Jan 6;41(1):BSR20193517. doi: 10.1042/BSR20193517 (PMC7789805; doi:10.1042/BSR20193517)
Supplement: Supplementary Tables S1-S2 [file BSR-2019-3517_supp.pdf]

Supplemental table 1. Downregulated 260 genes.

Gene Name	ENTREZ_GENE_ID	AFFY_ID
AGPAT3	56894	223182_S_AT
ABHD17A	81926	221267_S_AT
ASMTL	8623	36554_AT
ACTB	60	AFFX-HSAC07/X00351_M_AT<br>AFFX-HSAC07/X00351_5_AT
ACTA1	58	203872_AT
ACTC1	70	205132_AT
ACTG2	72	202274_AT
ACTN1	87	208637_X_AT
AP2S1	1175	208074_S_AT
ADD3	120	201753_S_AT
ADGRA2	25960	221814_AT
AKAP1	8165	201674_S_AT
AKAP8L	26993	218064_S_AT
ALDH3A2	224	210544_S_AT
ALKBH7	84266	223318_S_AT
ANKRD10-IT1	100505494	226663_AT
AAK1	22848	205434_S_AT
APOE	348	203381_S_AT/203382_S_AT
AQP5	362	213611_AT
ASS1	445	207076_S_AT
ABCC3	8714	230682_X_AT
ATP5G2	517	208764_S_AT
ATP2A3	489	207522_S_AT/213036_X_AT
ARID1A	8289	210649_S_AT/212152_X_AT
ATG16L2	89849	229389_AT
BCL6	604	228758_AT
BCL9L	283149	227616_AT
BMPR2	659	231873_AT
BAP1	8314	1555735_A_AT
BRD2	6046	208685_X_AT/214911_S_AT
BRD3	8019	203825_AT
BRD4	23476	202102_S_AT
BTG1	694	200920_S_AT
BTNL9	153579	229985_AT
CAMKK2	10645	213812_S_AT
CALD1	800	215199_AT
CNN1	1264	203951_AT
CA12	771	210735_S_AT/203963_AT
CARMN	728264	1558828_S_AT
COMP	1311	205713_S_AT
CSNK1A1	1452	240221_AT
CTSB	1508	227961_AT
CCZ1B	221960	215024_AT
CD248	57124	219025_AT
CD44	960	210916_S_AT
CD81	975	200675_AT
CD82	3732	203904_X_AT
CD99	4267	201028_S_AT
CLN6	54982	1567080_S_AT
CBX6	23466	202047_S_AT
CHD4	1108	201183_S_AT

C10orf10	11067	209183_S_AT
C12orf57	113246	224719_S_AT
CLOCK	9575	204980_AT
CFL1	1072	1555730_A_AT
CCDC85B	11007	204610_S_AT
COL1A2	1278	229218_AT
COL6A2	1292	209156_S_AT
COL7A1	1294	217312_S_AT
COL17A1	1308	204636_AT
CFDP1	10428	210701_AT
CTDSPL	10217	201904_S_AT
CUX1	1523	227069_AT
CXCL14	9547	237038_AT
CRIP2	1397	208978_AT
CYBA	1535	203028_S_AT
CYB5R3	1727	1554574_A_AT
DAB2	1601	201280_S_AT
DLEU2	8847	1556821_X_AT
DES	1674	214027_X_AT/202222_S_AT
DLG5	9231	210469_AT
DIXDC1	85458	214724_AT
DLGAP4	22839	1557394_AT
DMWD	1762	33768_AT
ENO1	2023	217294_S_AT
EGFR	1956	201983_S_AT
EIF4A1	1973	214805_AT
EIF5	1983	241843_AT
EXOSC7	23016	205200_AT
FAM107A	11170	209074_S_AT
FAM185A	222234	1556373_A_AT
FUBP3	8939	239193_AT
FASN	2194	212218_S_AT
FBXW5	54461	223050_S_AT
FBXO17	115290	220233_AT
FRMD4A	55691	1560031_AT
FTL	2512	213187_X_AT
FGFR1	2260	210973_S_AT
FNDC3B	64778	218618_S_AT
FLNA	2316	213746_S_AT
FLII	2314	222065_S_AT
FTX	100302692	1558515_AT
GNA11	2767	204248_AT
GALK2	2585	230918_AT
LGALS1	3956	201105_AT
LGALS7	3963	206400_AT
GATAD1	57798	214718_AT
GSN	2934	200696_S_AT
GTF3A	2971	238880_AT
GPX3	2878	214091_S_AT/201348_AT
GSTP1	2950	200824_AT
GAPDHP62	100128961	238807_AT
GAPDH	2597	AFFX-HUMGAPDH/M33197_5_AT<br>AFFX-HUMGAPDH/M33197_M_AT
GPSM2	29899	230002_AT

GAS6	2621	202177_AT/1598_G_AT
H1FX	8971	204805_S_AT
HEG1	57493	212822_AT
HSP90AB1	3326	214359_S_AT
HSPB7	27129	218934_S_AT
HGSNAT	138050	1557064_S_AT
HCRP1	387535	216176_AT
HIST2H2AA4	723790	214290_S_AT
IGFBP6	3489	203851_AT
IFIT3	3437	204747_AT
ISG20L2	81875	208114_S_AT
KLHL36	79786	238523_AT
KRT18	3875	201596_X_AT
KRT19	3880	201650_AT
KRT7	3855	209016_S_AT
KIFC3	3801	1559759_AT
KLF5	688	209212_S_AT
LARP1	23367	212193_S_AT
LMNA	4000	1554600_S_AT
LTBP3	4054	219922_S_AT
LTB4R	1241	216388_S_AT
LNPK	80856	225718_AT
LY6E	4061	202145_AT
LYRM9	201229	1560703_AT
KDM2A	22992	208987_S_AT
LAPTM5	7805	201721_S_AT
MIF	4282	217871_S_AT
HLA-B	3106	208729_X_AT/211911_X_AT
HLA-G	3135	211528_X_AT/210514_X_AT
MRGPRF	116535	227727_AT
MED13L	23389	216109_AT
MTHFR	4524	226929_AT
METTL26	84326	1554466_A_AT
METTL3	56339	242111_AT
MFAP2	4237	203417_AT
MIR6756	102465453	210869_S_AT
MIRLET7BHG	400931	232480_AT
MRPL12	6182	203931_S_AT
MRPL23	6150	213897_S_AT
MRPS18A	55168	221693_S_AT
MAP3K3	4215	203514_AT
MGLL	11343	211026_S_AT
MYH11	4629	201496_X_AT/201497_X_AT
MYO1C	4641	225080_AT/32811_AT/214656_X_AT
MYL9	10398	201058_S_AT
MYLK	4638	202555_S_AT
NDUFA11	126328	225304_S_AT
NBEAL2	23218	212443_AT
NF1P9	101930150	216115_AT
NTRK2	4915	221795_AT
NSUN6	221078	242239_AT
NAPA	8775	206491_S_AT
NFIX	4784	228278_AT

NPIPB5	100132247	215123_AT
NUCB1	4924	200649_AT
NDE1	54820	218414_S_AT
NUDT3	11165	212605_S_AT
OAZ1	4946	215952_S_AT
PXDN	7837	212013_AT
PEBP1	5037	211941_S_AT
PLD3	23646	201050_AT
PLPP3	8613	209355_S_AT/212226_S_AT
PYGB	5834	201481_S_AT
PIEZO1	9780	202771_AT
PKP3	11187	209873_S_AT
PABPN1	8106	213046_AT
PTRF	284119	1557938_S_AT/208790_S_AT
POU2F3	25833	215355_AT
PROSC	11212	209385_S_AT
P4HB	5034	200656_S_AT/1564494_S_AT
PSAP	5660	200866_S_AT
PTPN14	5784	244533_AT
PSMD6-AS2	100507062	232527_AT
PURA	5813	204021_S_AT
PDPR	55066	224902_AT
RABAC1	10567	203136_AT
RABL6	55684	225377_AT
RAB14	51552	211503_S_AT
RAB29	8934	218700_S_AT
RAD54L2	23132	213205_S_AT
RHOC	389	200885_AT
RDH5	5959	236291_AT
ARHGEF10	9639	1554703_AT
RNF145	153830	238949_AT
RNF213	57674	231956_AT/241347_AT
RNF216	54476	218425_AT
RNF24	11237	204669_S_AT
RNA45S5	100861532	225767_AT
RNF103-CHMP3	100526767	222437_S_AT
RRN3P3	100131998	238412_AT
RUNX3	864	204198_S_AT
RUNX1T1	862	205529_S_AT
SASH1	23328	41644_AT/213236_AT
SCAND1	51282	218206_X_AT
SFRP1	6422	202036_S_AT
SELENOM	140606	226051_AT
SENP3-EIF4A1	100533955	211787_S_AT/201530_X_AT
SRRM2	23524	208610_S_AT
SETD5	55209	221806_S_AT
SPPL3	121665	1554565_X_AT
STAT5B	6777	1555088_X_AT/1555086_AT
SRGAP1	57522	233888_S_AT
SNRPA1	6627	242146_AT
SNRPB	6628	213175_S_AT
SNORD139	116936	211666_X_AT
SNORA64	26784	217466_X_AT/212433_X_AT

SNORA68	26780	200869_AT
SLC25A6	293	212085_AT
SLC35E1	79939	235035_AT
SORBS2	8470	204288_S_AT
SP1	6667	224754_AT
SPRY1	10252	230212_AT
STARD10	10809	223103_AT
SOCS7	30837	226572_AT
SMARCA4	6597	212520_S_AT
SYMPK	8189	1554595_AT
SDC4	6385	202071_AT
STX16	8675	221499_S_AT
TUG1	55000	222244_S_AT
TBC1D5	9779	201813_S_AT
TNXA	7146	208609_S_AT/216333_X_AT
TSPAN4	7106	209264_S_AT
TGIF1	7050	1566901_AT
TXNIP	10628	201009_S_AT
TYMP	1890	204858_S_AT/217497_AT
TCF7L2	6934	212762_S_AT
TOB2	10766	221496_S_AT
TAGLN2	8407	200916_AT/210978_S_AT
TAGLN	6876	1555724_S_AT/205547_S_AT
TGOLN2	10618	212040_AT
TNPO2	30000	221507_AT
TRIM13	10206	229943_AT
TRIM29	23650	211002_S_AT/202504_AT
TRIM56	81844	231876_AT
TPP1	1200	214195_AT/214196_S_AT
UQCRC1	7384	201903_AT
USP13	8975	205356_AT
LOC100134445	100134445	236841_AT
UNK	85451	1562434_AT
URGCP	55665	244046_AT
UPK3BL	100134938	1552622_S_AT/1552621_AT
VGLL4	9686	214004_S_AT
VIM	7431	201426_S_AT
WASF2	10163	224562_AT
WNK1	65125	39313_AT
WISP2	8839	205792_AT
WWC1	23286	236725_AT
XAF1	54739	242234_AT
ZFP36L1	677	211965_AT
ZFP36L2	678	201369_S_AT
ZBTB16	7704	205883_AT
ZNF551	90233	211721_S_AT
ZNF652	22834	205594_AT
ZNF91	7644	206059_AT
ZKSCAN1	7586	214670_AT
ZNF638-IT1	100507113	239243_AT

Supplemental table 2. Upregulated 394 genes.

Gene Name	ENTREZ_GENE_ID	AFFY_ID
NT5DC1	221294	241962_AT
ABHD13	84945	234993_AT<br>235348_AT
ACPP	55	231711_AT
ADAM23	8745	244463_AT
AP1S3	130340	237159_X_AT
AP4S1	11154	210277_AT
ADGRF1	266977	236489_AT
ARL14	80117	220468_AT
ARL5B	221079	242727_AT
AKR1B10	57016	206561_S_AT
AGPS	8540	225114_AT
AREG	374	205239_AT
ANGEL2	90806	217630_AT
ANKRD13C	81573	1556361_S_AT
APELA	100506013	1559280_A_AT
APOL6	80830	1557236_AT
APOOL	139322	222269_AT
APLF	200558	241379_AT
ARG1	383	231662_AT
ARG2	384	203946_S_AT
ARMC8	25852	1555281_X_AT<br>1555279_AT
ABCA12	26154	215465_AT
ATP6V0E1	8992	214149_S_AT<br>214244_S_AT
ATP11A	23250	213582_AT
ATP11B	23200	1554557_AT<br>1564063_A_AT<br>1554556_A_AT
ATRX	546	208859_S_AT
ATG7	10533	1569827_AT
BHLHB9	80823	213709_AT
BLZF1	8548	32088_AT
BTF3L4	91408	235819_AT
BBIP1	92482	213220_AT
MCL1	4170	214056_AT
BCL2A1	597	205681_AT
B3GALT2	8707	210121_AT
BMP2K	55589	214716_AT
BOLA2	552900	231500_S_AT
BROX	148362	241908_AT
CDH26	60437	232306_AT
CALCRL	10203	234996_AT
CAPN14	440854	1557321_A_AT
CAPN6	827	202965_S_AT
CARD18	59082	231733_AT
CATSPER2	117155	1561405_S_AT
CATSPERB	79820	220293_AT
CCL13	6357	216714_AT
CCL20	6364	205476_AT
CD24	100133941	208650_S_AT
CD36	948	242197_X_AT
CEBPZOS	100505876	1564381_S_AT

CENPE	1062	205046_AT
CEP128	145508	244033_AT
CEP83	51134	239282_AT
CLCA4	22802	220026_AT
CHML	1122	1565951_S_AT
CHRNA9	55584	221107_AT
C1orf27	54953	222720_X_AT
C10orf99	387695	227736_AT
C12orf29	91298	228378_AT
C12orf4	57102	218374_S_AT
C12orf54	121273	240353_S_AT
C15orf48	84419	223484_AT
C15orf65	145788	243309_AT
C3orf33	285315	1554176_A_AT
C4orf47	441054	236915_AT
C7orf57	136288	1557636_A_AT
C9orf84	158401	233504_AT
CFAP97	57587	230569_AT
CKMT2-AS1	100131067	235443_AT
CLDND1	56650	239146_AT
CCDC186	55088	229399_AT
CCDC88A	55704	219387_AT
COX11	1353	214277_AT
CLEC5A	23601	219890_AT
CXCL17	284340	226960_AT
CXCL6	6372	206336_AT
CXCL8	3576	202859_X_AT
CDK1	983	231534_AT<br>203214_X_AT
DOCK9	23348	215237_AT
DEFB103A	414325	224239_AT
DEFB4B	100289462	207356_AT
DCK	1633	203302_AT
DEPDC1	55635	222958_S_AT
DSC2	1824	231033_AT
DUS3L	56931	230145_AT
DPH5	51611	222360_AT
DDIAS	220042	228281_AT
DNAJC21	134218	238336_S_AT<br>238335_AT
ELF5	2001	220625_S_AT
EEA1	8411	204840_S_AT
EGR1	1958	227404_S_AT
ENTPD6	955	234946_AT
EFCAB2	84288	211200_S_AT
ELAVL2	1993	228260_AT
ENKUR	219670	237314_AT
EREG	2069	205767_AT
EPB41L5	57669	230951_AT
ESF1	51575	222765_X_AT
ESCO1	114799	235645_AT<br>235216_AT
ESCO2	157570	235178_X_AT
ESR1	2099	205225_AT
EEF1D	1936	214395_X_AT

EEF1E1	9521	213907_AT
EIF5A2	56648	235296_AT
EIF5B	9669	214313_S_AT
EXOC5	10640	224253_AT
ESYT3	83850	1554912_AT
EYA3	2140	1554844_AT
FAM199X	139231	227133_AT
FAM200B	285550	227270_AT
FAM26F	441168	228362_S_AT<br>229391_S_AT
FAM84A	151354	234335_S_AT
FBXO30	84085	242007_AT
FBXO45	200933	242294_AT
FBN2	2201	203184_AT
FLG-AS1	339400	241014_AT
FOLH1B	219595	215363_X_AT<br>205860_X_AT<br>211303_X_AT
FRK	2444	207178_S_AT
GNB5	10681	242404_AT
GPR158	57512	232195_AT
GPR180	160897	1566256_S_AT<br>1556021_AT<br>1566257_AT
GABPB1-AS1	100129387	226900_AT
GLB1L3	112937	1569886_A_AT
GABRA4	2557	208463_AT
GGCX	2677	213705_AT
GJA3	2700	239572_AT
GTF2H1	2965	242656_AT
GLI4	2738	238364_X_AT
GLIPR1	11010	226142_AT
GUSBP4	375513	235888_AT
GK	2710	214681_AT<br>207387_S_AT
GPATCH2	55105	236026_AT
GDA	9615	224209_S_AT<br>1569555_AT
HOTS	103344718	224348_S_AT
HELZ	9931	240486_AT
HPSE	10855	219403_S_AT<br>222881_AT
HIST1H2BG	8339	215779_S_AT
HDAC8	55869	223908_AT
HCG18	414777	242618_AT
HYAL4	23553	220249_AT
HPGD	3248	211548_S_AT
IKZF2	22807	231929_AT
IDE	3416	217496_S_AT<br>203328_X_AT
IGF2BP3	10643	203819_S_AT<br>203820_S_AT
ITGB5	3693	214021_X_AT
IL1A	3552	210118_S_AT
IL19	29949	220745_AT
IL24	11009	206569_AT
IL36RN	26525	222223_S_AT
IL36G	56300	220322_AT
IFT74	80173	61732_R_AT
JMY	133746	241985_AT

KLK10	5655	215808_AT
KLK13	26085	205783_AT
KLK6	5653	204733_AT
KANSL1-AS1	644246	229857_S_AT
KLHL41	10324	219106_S_AT
KLHL7	55975	220239_AT<br>220238_S_AT
KBTBD8	84541	239835_AT
KIAA1586	57691	231869_AT
KLRB1	3820	214470_AT
KLRC1	3821	206785_S_AT
KNL1	57082	228323_AT
KRAS	3845	204010_S_AT
KRIT1	889	229785_AT
KYNU	8942	217388_S_AT
LARP4	113251	214155_S_AT
LEF1-AS1	641518	243362_S_AT
LMTK2	22853	235307_AT
LIMS3-LOC440895	100271835	229095_S_AT
LIN7C	55327	219399_AT
LINS1	55180	220121_AT
LIPG	9388	219181_AT
LINC01094	100505702	229635_AT
LINC01215	101929623	236198_AT
LINC01578	100507217	230156_X_AT<br>226830_X_AT
LINC00302	388699	216718_AT<br>216935_AT
LINC00964	157381	236950_S_AT
KDM7A	80853	225142_AT
MACC1	346389	1566764_AT<br>232151_AT
MAGT1	84061	210596_AT
MDH1B	130752	1558077_S_AT
MAP7D2	256714	228262_AT
MMP16	4325	223614_AT
MXD1	4084	228846_AT
MND1	84057	223700_AT
MS4A1	931	228592_AT
MS4A4A	51338	219607_S_AT
MALAT1	378938	226675_S_AT
MTHFD2L	441024	220346_AT
METTL10	399818	226634_AT
METTL6	131965	1553689_S_AT
MIR17	406952	232291_AT
MSMB	4477	207430_S_AT
MIER3	166968	231975_S_AT
MCM9	254394	1553759_AT
MCM6	4175	238977_AT
MTFMT	123263	235689_AT
MRPL45P2	653479	237182_AT
MTRF1	9617	242996_AT
MKX	283078	239468_AT
MPHOSPH6	10200	1554906_A_AT
MYO10	4651	1554026_A_AT
MTMR6	9107	228789_AT

NAA15	80155	222837_S_AT
NADK2	133686	229299_AT
ND6	4541	1553575_AT
NCK1	4690	244487_AT
N4BP2	55728	228242_AT
NRG4	145957	242426_AT
NBEAL1	65065	1561589_A_AT
NEFH	4744	33767_AT
NAV3	89795	1552658_A_AT
NME8	51314	220384_AT
NCAPG	64151	218662_S_AT
NOP14-AS1	317648	214123_S_AT
NOP16	51491	209327_S_AT
NSMCE4A	54780	227387_AT
NUP160	23279	214963_AT
NUP58	9818	225047_AT
NUDCD1	84955	225438_AT
NUDCD2	134492	226643_S_AT
ORC4	5000	203352_AT
OGN	4969	218730_S_AT
OSTM1	28962	235197_S_AT
OTUD1	220213	231035_S_AT
PACRGL	133015	235554_X_AT
PATJ	10207	240570_AT
PDLIM4	8572	214174_S_AT
PI3	5266	203691_AT
PEX13	5194	1558164_S_AT
PPARD	5467	242218_AT
PHF14	9678	204525_AT
PIGG	54872	231692_AT
PIGX	54965	227403_AT
PGM2	55276	225366_AT
PLCD4	84812	224505_S_AT
PSPH	5723	205048_S_AT
PCLO	27445	213558_AT
PLAC8L1	153770	237783_AT
PLAG1	5324	205372_AT
PLEKHA5	54477	233040_AT
PHLDA2	7262	229494_S_AT
PLK4	10733	204886_AT
PABPC4L	132430	238865_AT
KCMF1	56888	242887_AT
KCTD4	386618	239787_AT<br>240512_X_AT
PFDN4	5203	205362_S_AT
PRELID3B	51012	229835_S_AT
POF1B	79983	1555383_A_AT<br>219756_S_AT
PPBP	5473	214146_S_AT
PTGS2	5743	204748_AT
PRSS27	83886	232074_AT
PKIA	5569	226864_AT
PPP3R1	5534	204507_S_AT
PPM1A	5494	231370_AT<br>229027_AT

PPM1L	151742	229506_AT
PTPN4	5775	230230_AT
PCDH17	27253	228863_AT
PCP4L1	654790	241382_AT
RAB27B	5874	207018_S_AT
RALGPS2	55103	242458_AT
RAP2A	5911	1556809_A_AT
RASEF	158158	235144_AT
RIT1	6016	236223_S_AT
RGS1	5996	202988_S_AT
RGS10	6001	214000_S_AT
RGS13	6003	1568752_S_AT
RGS18	64407	223809_AT
RGS20	8601	210138_AT
RPTN	126638	1553454_AT
RIF1	55183	214700_X_AT
ARHGAP11A	9824	204492_AT
ARHGEF12	23365	233621_S_AT
RPS10	6204	214001_X_AT
RNMT	8731	202684_S_AT
POLR3G	10622	206653_AT
RPARP-AS1	100505761	213964_X_AT
S100A12	6283	205863_AT
S100A7A	338324	232170_AT<br>232220_AT
S100A8	6279	214370_AT
SAMSN1	64092	220330_S_AT
SAP30L	79685	226772_S_AT
SELENOI	85465	1555274_A_AT
LOC645513	645513	1561760_S_AT<br>1561761_X_AT<br>1561759_AT
LOC645513	645513	1561759_AT
LOC645513	645513	1561761_X_AT
SPINK6	404203	1553973_A_AT
SPINK7	84651	223720_AT
STYX	6815	235180_AT
STYK1	55359	220030_AT
SERPINB3	6317	209719_X_AT
SETDB2	83852	235338_S_AT
SH3BP2	6452	217257_AT
SPCS3	60559	218817_AT
STAM2	10254	215044_S_AT
SIM1	6492	1556300_S_AT
SMIM17	147670	1557430_AT
SNHG10	283596	244786_AT
SNORD116-4	100033416	1559343_AT
SNORA21	619505	214744_S_AT
SPRR2B	6701	208539_X_AT
SPRR2C	6702	220664_AT
SPRR3	6707	218990_S_AT<br>232082_X_AT
SLC16A1	6566	202235_AT
SLC16A10	117247	236220_AT
SLC16A7	9194	241866_AT
SLC28A3	64078	232277_AT

SLC44A5	204962	235763_AT
SLC6A14	11254	219795_AT
SLC7A11	23657	209921_AT
SPAG8	26206	206816_S_AT
SPATA6L	55064	1554708_S_AT
SGMS2	166929	242963_AT
SKA2	348235	225686_AT
SPON1	10418	213993_AT
SPRTN	83932	223511_AT
ST8SIA1	6489	1569788_AT
SBNO1	55206	218737_AT
SMC3	9126	209258_S_AT
STX17-AS1	441461	1558689_A_AT
SULT1E1	6783	222940_AT
SENP5	205564	57703_AT
ST7L	54879	1552738_A_AT
SIKE1	80143	204666_S_AT
STX19	415117	1555173_AT
TRAT1	50852	217147_S_AT
TH2LCRR	101927761	1563389_AT
TAC1	6863	206552_S_AT
TC2N	123036	1553132_A_AT
TAS2R14	50840	235762_AT
TAF7L	54457	220325_AT
TEX10	54881	1558702_AT
TEX101	83639	223906_S_AT
TESMIN	9633	238246_AT
TTC39B	158219	236826_AT
TAB3	257397	1552928_S_AT
THAP9	79725	230185_AT
TXNDC9	10190	1554047_AT
TXN	7295	216609_AT
TIGD1	200765	1553099_AT
TNNI3K	51086	241858_AT
TIGAR	57103	219099_AT
TRAF3IP3	80342	240265_AT
TRAPPC11	60684	234747_AT
TCN1	6947	205513_AT
TIMM17A	10440	201822_AT
TMPRSS11D	9407	207602_AT
TMEM170B	100113407	235798_AT
TMEM45B	120224	230323_S_AT
TMEM67	91147	238229_AT
TMEM70	54968	226083_AT
TMEFF2	23671	224321_AT
TRIM10	10107	221627_AT
TRIM13	10206	240288_AT
TYW5	129450	235181_AT
TMOD3	29766	220800_S_AT
TTC28-AS1	284900	244189_AT
UBASH3B	84959	238462_AT
UBE2W	55284	241887_AT
UBR3	130507	244121_AT

UGCG	7357	221765_AT
LOC100130691	100130691	231540_AT
LOC100505555	100505555	1569156_AT
LOC100506498	100506498	1557921_S_AT
LOC100507431	100507431	1552972_AT
LOC101927972	101927972	240361_AT
LOC101928100	101928100	242873_AT
VPS13A	23230	231585_AT
VNN1	8876	205844_AT
VNN3	55350	220528_AT
VSNL1	7447	203798_S_AT
WDHD1	11169	216228_S_AT
WDR37	22884	242255_AT
WDR78	79819	1554140_AT
XCL1	6375	214567_S_AT
YIPF6	286451	212342_AT
YOD1	55432	215150_AT
ZBTB43	23099	231393_X_AT
ZDHHC21	340481	235068_AT<br>243550_AT
ZFYVE16	9765	1555982_AT
ZNF101	94039	1552634_A_AT
ZNF165	7718	206683_AT
ZNF197	10168	233070_AT
ZNF585A	199704	227674_AT
ZNF621	285268	1558620_AT
ZNF678	339500	242923_AT
ZNF681	148213	238962_AT
ZNF709	163051	1553247_A_AT
ZNF711	7552	207781_S_AT
ZNF782	158431	235290_AT
ZNF891	101060200	1569283_AT
ZNF529-AS1	101927599	237364_AT
ZNF674-AS1	401588	241972_AT
ZYG11A	440590	231517_AT
